# Increasing Screening Rates for Comorbidities in Adolescents with Elevated Body Mass Index in Pediatric Primary Care

**DOI:** 10.1097/pq9.0000000000000747

**Published:** 2024-07-10

**Authors:** David R. Karas, Sharon Juszli, Marnie Walston, April Love, Michael T. Bigham

**Affiliations:** From the * Department of Pediatrics, Akron Children’s Hospital, Akron, Ohio; † Northeast Ohio Medical University, Rootstown, Ohio.

## Abstract

**Introduction:**

Adolescents with elevated body mass index are at increased risk for comorbidities such as dyslipidemia, diabetes mellitus, and metabolic dysfunction-associated steatotic liver disease. Guideline-based screening can identify impacted patients early, allowing for lifestyle modifications and other treatments to improve long-term health. Unfortunately, only 20% of pediatric patients with obesity receive recommended screening.

**Methods:**

A multidisciplinary quality improvement team designed and implemented a project to improve comorbidity screening utilizing the Model for Improvement. Provider education and incentive, clinical decision support, and regular performance feedback were chosen as interventions. Screening rates were tracked on a statistical process control chart.

**Results:**

From March through December of 2022, 9547 pediatric patients aged 10 years and up with body mass index greater than or equal to the 95^th^ percentile were seen for preventive care visits. Screening rates for comorbidities increased from a baseline of 19.5%–58% and were sustained for over 3 months. Numerous patients at risk for chronic disease were identified.

**Conclusions:**

Evidence-based clinical decision support, along with provider education and engagement, can effectively increase screening rates for comorbidities in pediatric patients with obesity.

## INTRODUCTION

Obesity is the most common chronic disease of childhood in the United States, affecting more than 14.7 million children and adolescents.^1^ Defined as a body mass index (BMI) at or above the 95^th^ percentile of the Centers for Disease Control sex-specific BMI-for-age growth charts, obesity has complex physiologic, genetic, socioeconomic, and environmental causes. Obesity is preventable and treatable but tends to relapse, and children with obesity are five times more likely to become adults with obesity when compared with their healthy-weight peers.^2^ Obesity can lead to increased risk for many serious diseases and health problems, including high blood pressure, high cholesterol, type 2 diabetes, sleep apnea, liver disease, heart disease, and stroke, and severe obesity may shorten life expectancy by up to 14 years.^3^ The financial cost of obesity is also significant. In 2016, the cumulative medical cost due to obesity among adults in the United States was $260.6 billion.^4^ These concerns have become even more relevant as the rate of obesity among adults, adolescents, and children in the United States has increased since the beginning of the COVID-19 pandemic.^5^ In just the first year of the pandemic, the percentage of 12 to 15-year-olds with overweight and obesity increased by over five percentage points.^6^

In 2023, the American Academy of Pediatrics published a clinical practice guideline on evaluating and treating pediatric obesity.^7^ The guideline outlines a strategy for laboratory evaluation to identify common obesity-related conditions such as prediabetes and type 2 diabetes, dyslipidemia, and metabolic dysfunction-associated steatotic liver disease (formerly known as nonalcoholic fatty liver disease), all of which have increased prevalence in children and adolescents with obesity.^8^ Early detection of these comorbidities can guide treatment, reduce risk factors, and motivate patient engagement. A fasting lipid panel is the recommended screening test for evaluating lipid abnormalities. Acceptable diagnostic tests for prediabetes and diabetes include fasting plasma glucose, 2-hour plasma glucose after oral glucose tolerance test, and hemoglobin A1c. The recommended screening test for evaluation of metabolic dysfunction-associated steatotic liver disease (MASLD) is alanine transaminase (ALT). Testing should be repeated in 2 years if results are normal. In a recent national cohort study, adherence with recommended laboratory screening for comorbid medical conditions associated with obesity was found to be no higher than 65% in pediatric primary care settings, and only one in five children received the complete bundle of recommended screening laboratories for hyperlipidemia, MASLD, and type 2 diabetes.^9^

Locally, a 2020 retrospective chart review showed that the bundle of screening laboratories was ordered 19% of the time during well-child visits for children 10 years and older with a BMI at or above the 95^th^ percentile in the primary care offices of a freestanding pediatric health system in the Midwestern United States. To improve knowledge and compliance with recommended screening for children with elevated BMI, a quality improvement (QI) project was conducted in 2020 at five pilot offices (n = 23 providers). Participant chart reviews showed that 25% (n = 316) of patients 10 years or older with a BMI at or above the 95^th^ percentile (N = 1248) received recommended screening laboratories from January through September 2020. A narrated electronic education module paired with periodic compliance reporting improved screening rates for obese children to 39% during the intervention months (October 2020–January 2021). Pilot practices sustainably screened at a higher rate for 3 months after the intervention period, demonstrating a successful change in practice warranting project spread.^10^

In 2021, our organization saw over 48,000 children ages 10 through 21 for preventive care visits, and 23% had a BMI at or above the 95^th^ percentile. The recommended screening tests were not ordered for nearly 80% of those patients with obesity. The improvement aim was to increase the percentage of preventive care patients 10 through 21 years with a BMI ≥ 95^th^ percentile for whom screening laboratories for diabetes mellitus, MASLD, and dyslipidemia were ordered if not done in the past 2 years from 19.5% to 50% by the conclusion of the project. This QI project was exempt from institutional review board oversight due to the nonexperimental nature of the work.

## METHODS

### Setting and Improvement Team

**Fig. 1.**
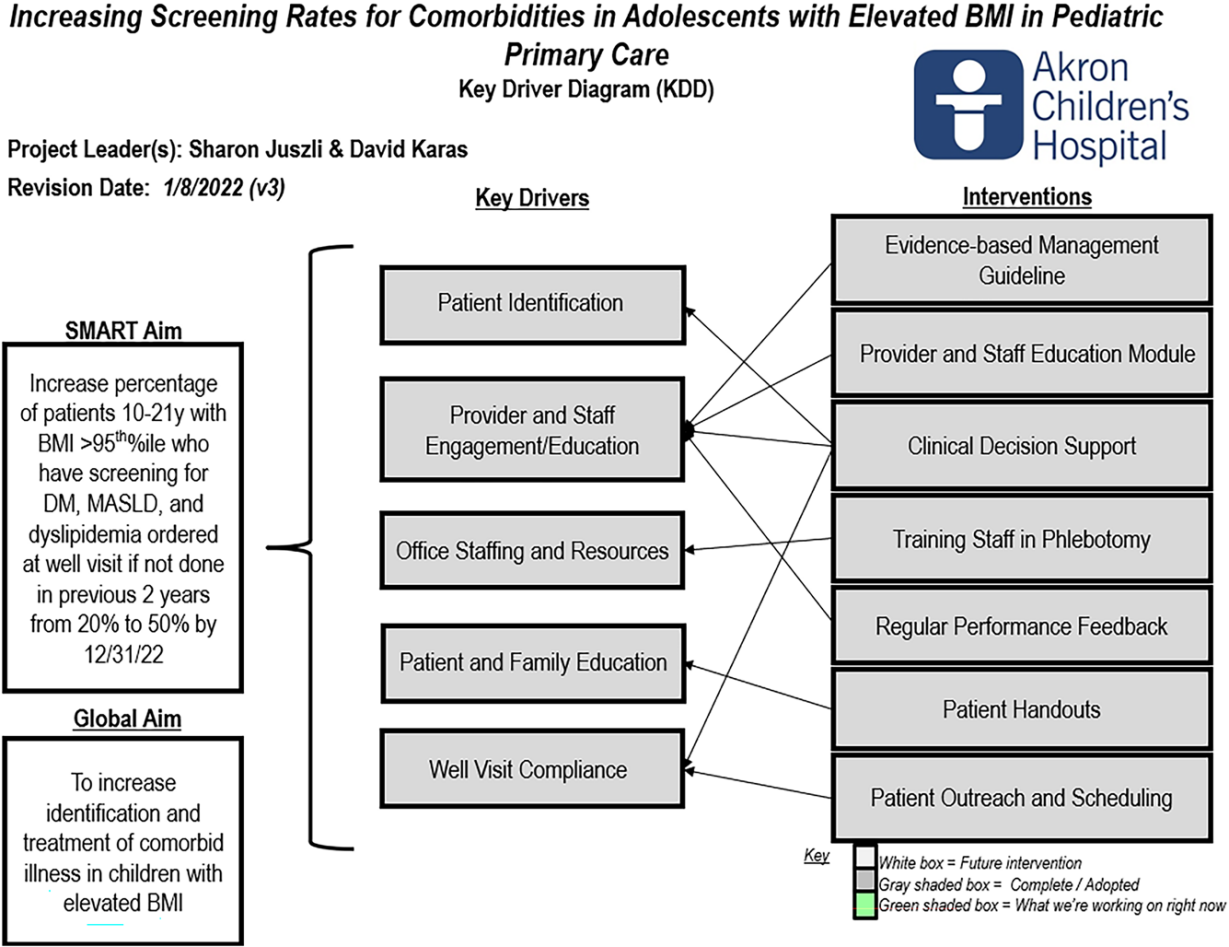
Key driver diagram demonstrating global aim, SMART aim, the target population, key drivers, and interventions necessary to achieve SMART aim.

Akron Children’s Hospital has a network of 37 primary care locations across 15 counties, with a total panel size of over 220,000 patients and over 523,000 visits annually. Informed by baseline 2021 data on the management of pediatric obesity derived from our shared EHR (Epic Systems Corp, Verona, Wisc.), we assembled a multidisciplinary improvement team. The improvement team included primary care pediatricians, a pediatric obesity medicine physician, a doctorate-prepared pediatric nurse practitioner, clinical informaticists, and electronic health record (EHR) analysts. The team utilized the Model for Improvement to guide the work.^11^ After identifying multiple key drivers (Figure 1), the team chose interventions to test that focused on provider education, the development of EHR-based clinical decision support (CDS) tools, and provider engagement using feedback and incentives.

### Interventions

#### Education

An evidence-based, narrated electronic slide deck informed providers and staff on the clinical practice guidelines for children with elevated BMI (**Supplemental Digital Content,** http://links.lww.com/PQ9/A571). This one-time training was required for all project participants. The presentation included information regarding the prevalence of pediatric overweight and obesity; the association between elevated BMI and comorbid illness; recommended laboratory testing for children with overweight and obesity; compliance with screening guidelines found in the literature and locally, benefits of compliance, and how to use order sets for elevated BMI in the EHR. The education module included links to download evidence-based practice guideline algorithms to help manage abnormal laboratory results and a referral guide for comorbid illnesses. Patient and family education packets were provided to each office for providers to use for teaching. They included handouts on healthy breakfast and snack options (Academy of Nutrition and Dietetics), portion education (MyPlate.gov), 5-2-1-0 for a Healthier Lifestyle, and BMI Red Zone charts for boys and girls (created by the Healthy Active Living Program at this institution).

#### Clinical Decision Support

**Fig. 2.**
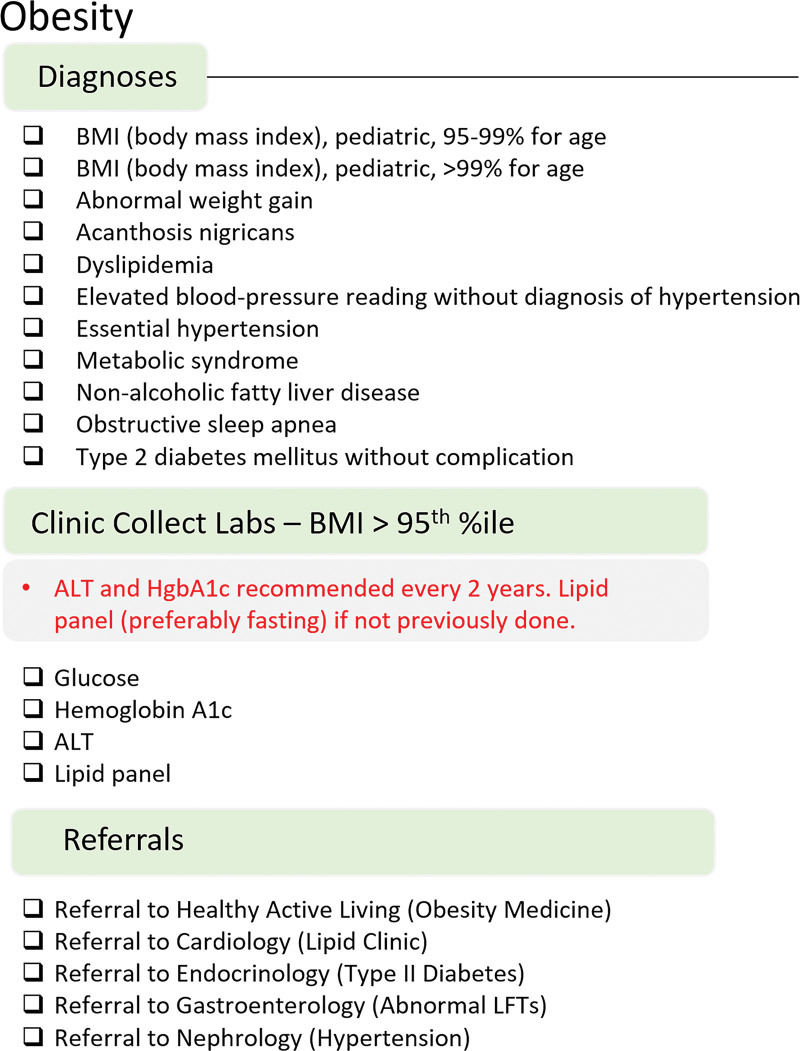
Obesity order set showing common diagnoses, CDS, laboratory orders, and referrals.

The EHR allows for the creation of standardized order sets to improve provider efficiency and adherence to evidence-based recommendations.^12^ Order sets group relevant diagnoses, laboratory tests, imaging orders, medications, and follow-up appointments in one location. Order sets can be suggested based on the patient’s chief complaint, chronic medical problems, abnormal screening results, and presenting vital signs. Our organization previously had a suite of CDS tools to help guide the diagnosis and management of overweight and obesity. A pop-up advisory suggests adding the appropriate diagnosis to the problem list for patients with an elevated BMI. For patients two years and up with elevated BMI on their problem list, an obesity order set is suggested. The dynamic order set displays guideline-based recommendations based on the patient’s age and BMI (Fig. 2). We updated the order set with a better list of diagnoses, more concise CDS, and clearer referral recommendations.

#### Provider Engagement

Provider engagement was achieved using data transparency, certification credit, and incentive compensation dollars. Primary care providers received monthly reports on individual, peer, and office ordering rates of BMI screening laboratories for eligible patients. These were reported using nonanonymized ranked lists for individual providers’ performance and statistical process control charts for aggregate performance. Providers enrolled in the American Board of Pediatrics Maintenance of Certification program could obtain Part 4 credit. Additional incentives for providers included a financial bonus payment for those who screened more than 40% of qualifying patients during the study period. Although the project goal was 50%, we wanted to reward all providers who demonstrated meaningful improvement.

### Measurement

The primary outcome measure was the percentage of patients 10 years and older presenting for preventative care visits with a BMI ≥ 95^th^ percentile who received the bundle of recommended screening laboratory orders if not completed in the last 2 years. The recommended screening laboratory orders included a lipid panel, ALT, and hemoglobin A1c or fasting blood glucose. Screening order rates were tracked on a statistical process control chart, and standard control chart rules identified special cause variation. A secondary measure included the percentage rate of abnormal laboratories in patients for whom results were available. Additionally, we measured referral rates to key specialties during the study period and for the same time frame 1 year prior.

## RESULTS

**Fig. 3.**
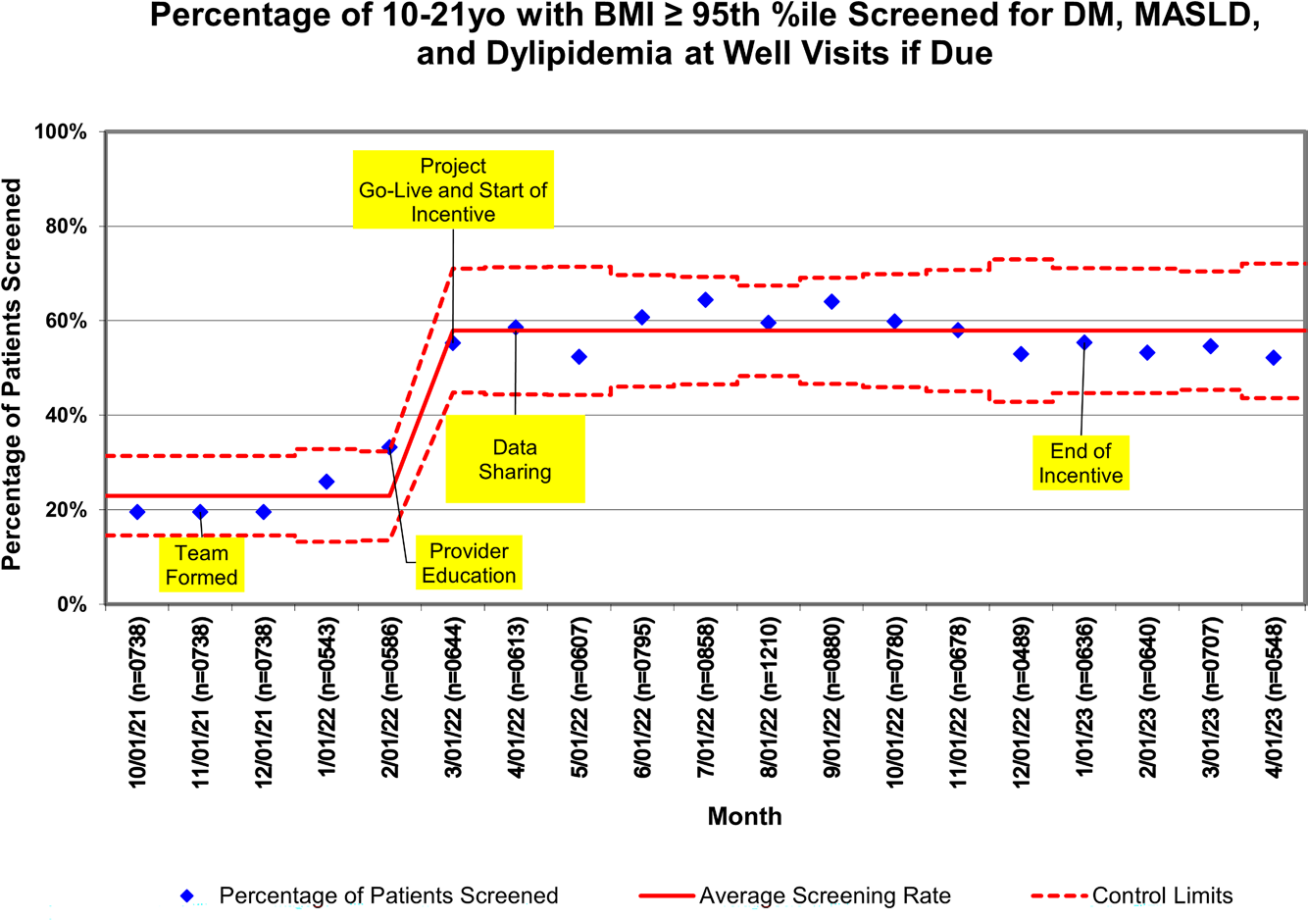
P prime statistical process control chart depicting the percentage of preventive care patients appropriately screened. Annotations identify the timing of key interventions. The solid red line is the mean screening rate, and the dashed lines are the monthly control limits.

Between March 1, 2022, and December 31, 2022, there were 42,467 preventative care visits for 10-year and older patients. Nine thousand five hundred forty-seven patients (22.5%) had a BMI ≥ 95^th^ percentile. Of these, 7554 (79.1%) had not been screened for comorbidities in the past 2 years. The demographics of patients with BMIs below and above the 95^th^ percentile are described in Table 1. Black children, Hispanic children, and those with public insurance were all overrepresented in the elevated BMI group. The historical baseline screening laboratory order rate at well visits was 19.5%. After providing education and regular feedback, we increased our bundled screening rate to 58%. The statistical process control chart can be found in Figure 3.

**Table 1.** Demographics of Patients 10 Years and Up Presenting for Preventive Care during the Study Period by BMI Percentile

	BMI < 95^th^ %ile n = 32,920	BMI ≥ 95^th^ %ile N = 9547
Male	16,131 (49.0%)	5,050 (52.9%)
Female	16,789 (51.0%)	4497 (47.1%)
Black	4642 (14.1%)	2014 (21.1%)
White	26,599 (80.8%)	7256 (76.0%)
Hispanic	1086 (3.3%)	477 (5.0%)
Non-Hispanic	30,583 (92.9%)	8764 (91.8%)
Public insurance	12,147 (36.9%)	5251 (55.0%)

Although not designed to determine the prevalence of specific diseases, available results depict a population at risk. 11.5% of patients had an elevated ALT. Non-HDL cholesterol was outside of the normal range in 39.8%. Hemoglobin A1c results indicated that 1.2% of children were diabetic and 22.1% had prediabetes. At least nine adolescents with previously undiagnosed diabetes mellitus were identified.

We also tracked referrals to specific specialties. Table 2 shows the project’s impact on referral rates for obese preventive care patients.

**Table 2.** Percentage of Preventive Care Patients 10 Years and Up with BMI Greater Than or Equal to the 95^th^ Percentile Referred to Pediatric Specialties by Year

	2021 Percentage of Patients Referred n = 9521	2022 Percentage of Patients Referred n = 9411	Change in Percentage
Obesity medicine	6.4%	6.0%	−0.4
Endocrinology	2.1%	2.0%	−0.1
Gastroenterology	2.6%	2.2%	−0.4
Cardiology	2.9%	2.6%	−0.3

## DISCUSSION

This project successfully improved adherence to all laboratory screening recommendations for primary care patients with obesity from 19.5% to 58% during the improvement project. This exceeded the national rate of 20% for comorbidity screening.^9^ We accomplished this by prioritizing the project on a departmental level, committing resources and focus to the project, educating providers on the scope and severity of the problem, and leveraging CDS tools in the EHR. Providing individual feedback and several incentives to providers contributed to the project’s ultimate success. This improvement effort demonstrates the repeatability of a successful and sustainable improvement methodology across a large primary care network. Previous work has demonstrated that a suite of similar interventions can improve other domains of primary care screening and clinical management.^13,14^

We expected that improved adherence to laboratory screening recommendations for patients with obesity might have had a secondary outcome of increasing referrals to subspecialties, including endocrinology, cardiology, gastroenterology, and obesity medicine. This was not the case, however. Referrals to cardiology and gastroenterology were lower in the intervention year than in the prior year. Increased screening identified numerous patients with abnormal glucose regulation, but referrals to endocrinology did not increase compared with the previous year. This may be because embedded within the education module were linked evidence-based practice guideline algorithms for providers to download. These guidelines listed the frequency of repeat abnormal laboratories and the thresholds for which a specialty referral was indicated. Providers were encouraged to send patients with mild laboratory abnormalities to the multidisciplinary obesity medicine clinic rather than to the other specialties. Surprisingly, referrals to the obesity medicine clinic also did not increase over the prior year. This may indicate that abnormal laboratory results do not affect provider decision-making on who to refer for comprehensive obesity treatment. It is possible that most referrals to the obesity management clinic occur at the time of the provider visit and before receiving results from the laboratory tests. It also may reflect that healthcare providers generally lack confidence in the interventions available for the management of obesity, locally resulting in the referral of only 6.4% of patients for treatment despite ordering screening laboratories and presumably discussing the diagnosis with at least 59% of patients with obesity.^15^ This perspective is supported by qualitative research investigating the challenges accompanying the management of obesity. Additionally, educational discussion topics on lifestyle changes and nutrition handouts were made available for providers. Perhaps providers were empowered by these education tools to manage abnormal laboratory results in the primary care office, leading to stable specialty referrals despite increased screening and abnormal screening results.

Another possible secondary outcome of this project is that adherence to laboratory screening recommendations for patients with obesity may have forced uncomfortable discussions of weight and elevated BMI on primary care providers who are not accustomed to having these sensitive conversations. The organization’s qualitative feedback from patients and families indicated an increase in negative comments related to how providers addressed issues of elevated weight or BMI during their appointments. Literature supports that families often do not want to discuss weight at their first meeting with a medical provider and prefer to have a relationship first. However, to comply with the quality project, providers often needed to discuss weight and order laboratories at their first meeting with a patient.^16^ Providers did not receive specific training on weight stigma or language surrounding obesity, which may have resulted in clinicians inadvertently using language deemed offensive by patients and families. For example, parents of children with overweight and obesity report preferring the term “gaining too much weight” over “overweight,” “fat,” and “obese,” and adolescents with obesity prefer that providers use the terms “weight,” “weight problem” and “plus size” when discussing body weight rather than using the terms “obese,” “large” or “fat.” Avoiding stigmatizing language is important for preserving the doctor–patient relationship and engaging patients in care, as 37% of parents would feel upset and embarrassed, and 24% would avoid future appointments if they felt their child was stigmatized about their weight by a healthcare provider.^17^ Healthcare providers report being equally concerned with offending patients and potentially jeopardizing the doctor-patient relationship by raising concerns about obesity. So, before the QI project, providers may have avoided the subject entirely.^15^

Screening for comorbidities is just a small piece of the management of patients with overweight and obesity. Satti et al improved laboratory testing, counseling, and referrals for some patient groups through a combination of provider education and clinical decision support.^18^ Chronic diseases require longitudinal care, so other groups have focused on follow-up. One group more than tripled the number of patients who returned for follow-up by using “prescriptions” for weight management visits.^19^ EHR-based CDS is a common theme in the literature and offers the hope that standardization of treatment according to guidelines can lead to improved outcomes.

## LIMITATIONS

This improvement project does have some limitations. This work focused on children receiving care at well-child visits, excluding those seen in ambulatory or inpatient settings outside of the primary care practice and those seen at the primary care practice for sick visits. The project did not measure order completion. although most laboratories are drawn on-site, over 15% of families had testing done outside our system, and discrete results were unavailable. This study did not track long-term health outcomes for patients who received appropriate screening orders, as this was beyond the scope of the study. Another limitation is that when we quantified referrals, we did not measure the completion of those visits or identify the reason for the referral. Finally, the study focused only on screening recommendations for patients 10 years and older with a BMI ≥ 95th percentile. However, there are also obesity laboratory screening recommendations for patients < 10 years of age and those with a BMI in the overweight category, between the 85th and 95th percentile.

## CONCLUSIONS

This study illustrates a successful increase in adherence to recommendations for obesity screening laboratories for children 10 years of age and older with a BMI ≥ 95th percentile using a multidisciplinary improvement team, the Model for Improvement methodology, and the EHR and CDS systems. Healthcare organizations can use similar CDS tools to improve screening for obesity-related comorbidities. In the future, we will seek to improve our systems for tracking the completion of ordered laboratory tests and managing children with abnormal results on obesity screening laboratories.

## Supplementary Material


