# Supplementary material for: Increasing Screening Rates for Comorbidities in Adolescents with Elevated Body Mass Index in Pediatric Primary Care
Source: Pediatr Qual Saf. 2024 Jul 10;9(4):e747. doi: 10.1097/pq9.0000000000000747 (PMC11236401; doi:10.1097/pq9.0000000000000747)
Supplement: Supplementary file 1 [file pqs-9-e747-s001.pdf]

Quality Improvement Project to Improve Lab Screening for Children with Elevated BMI

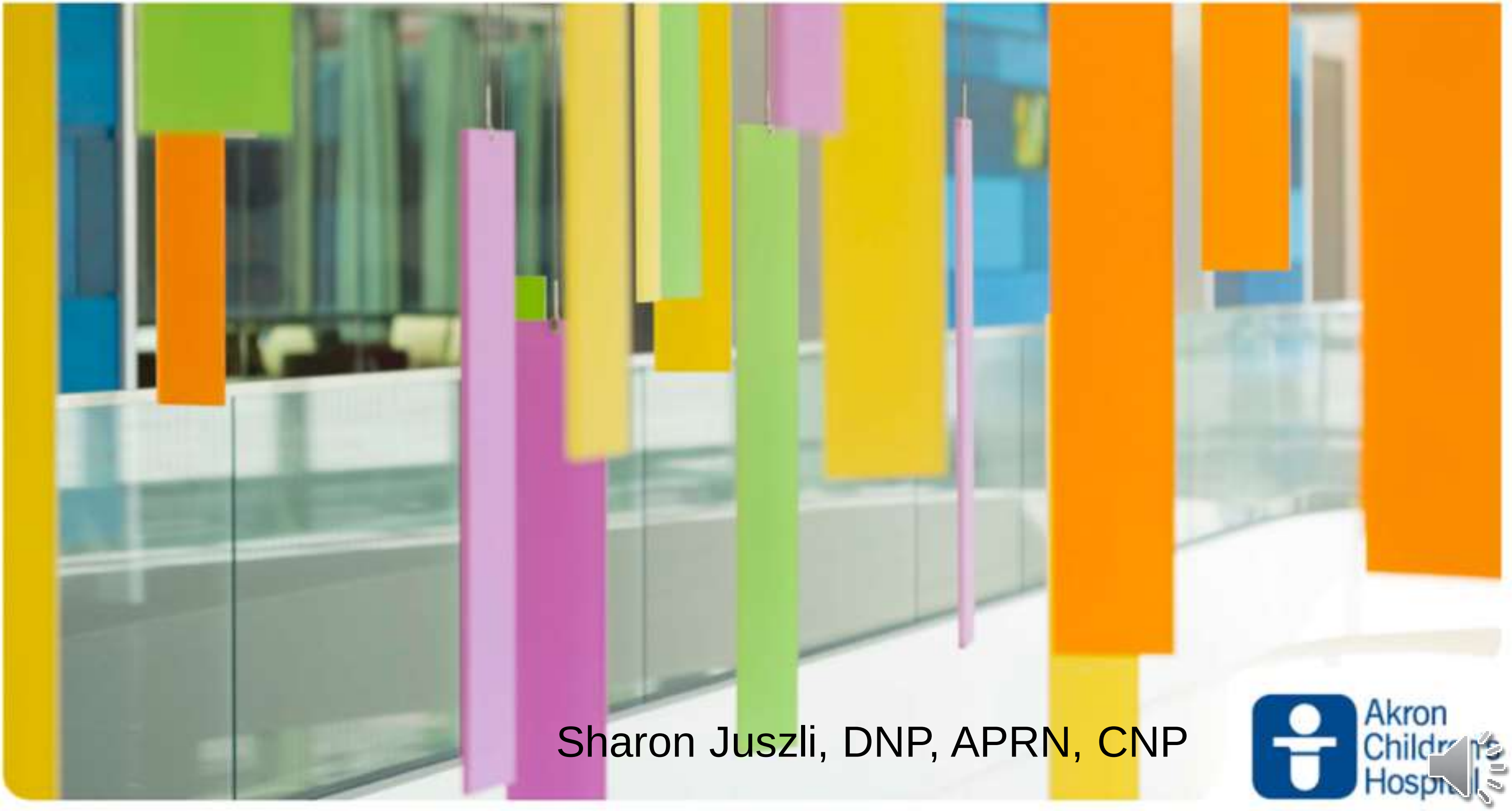

Sharon Juszli, DNP, APRN, CNP

Akron Children's Hospital

Pretest

- Click here

Growth Chart for Boy 2-20 Years

Height

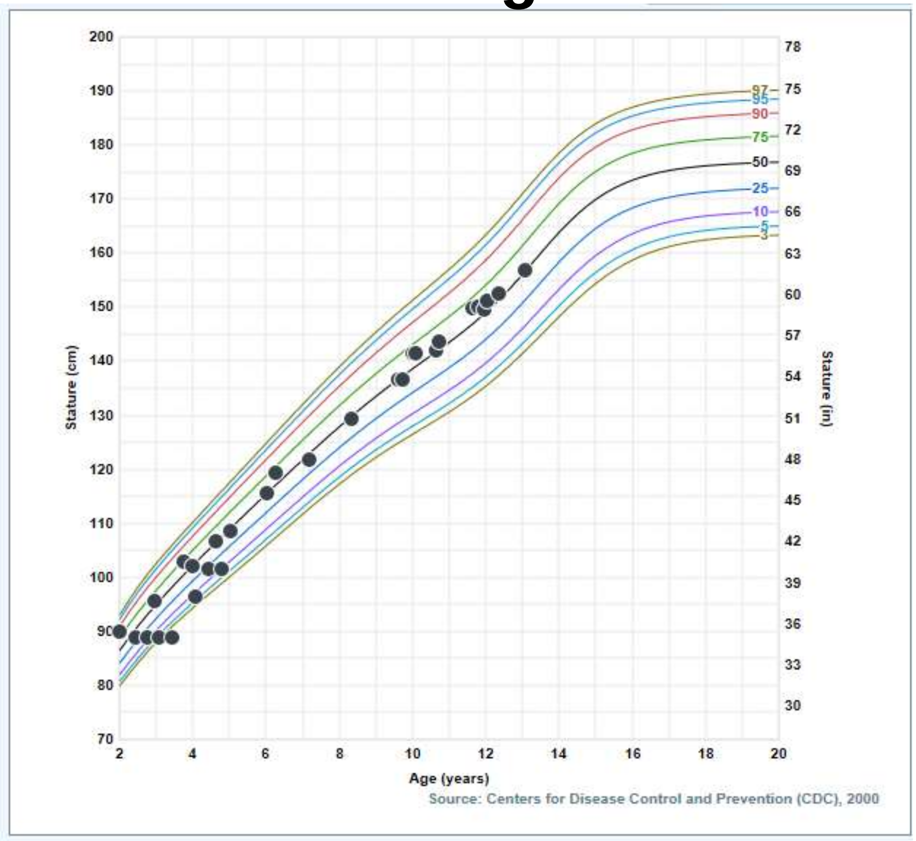

Weight

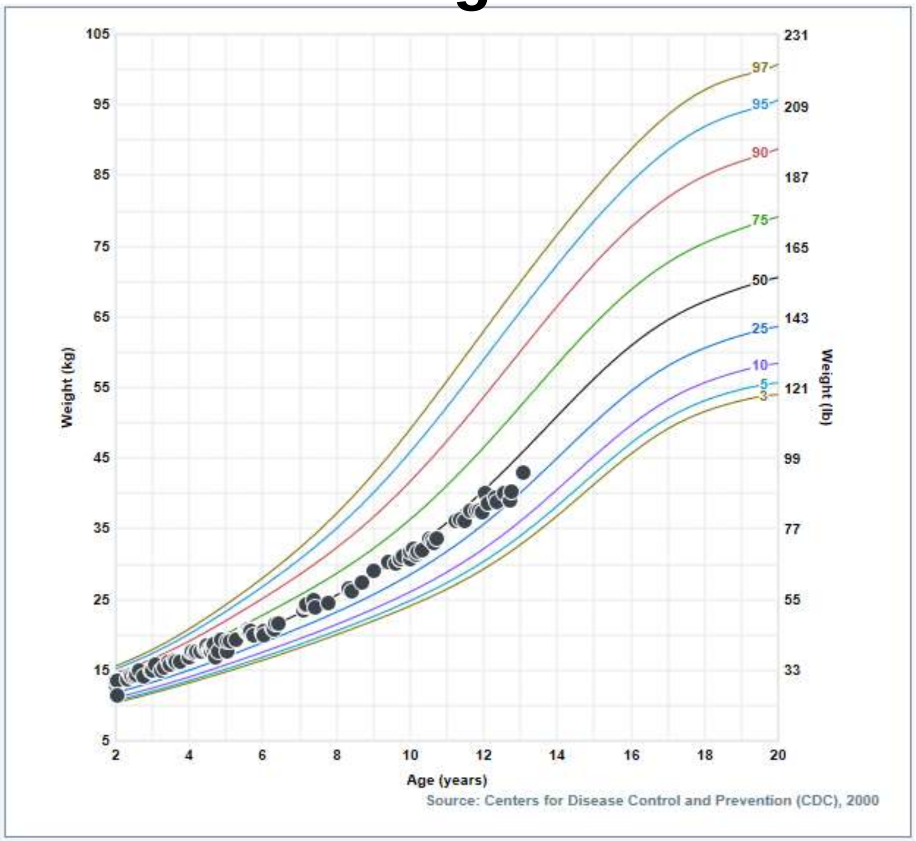

(CDC, 2000)

Body Mass Index (BMI)

HOW TO CALCULATE BODY MASS INDEX

$$BMI = \frac{Weight\ (kg)}{[Height(m)]^2}$$

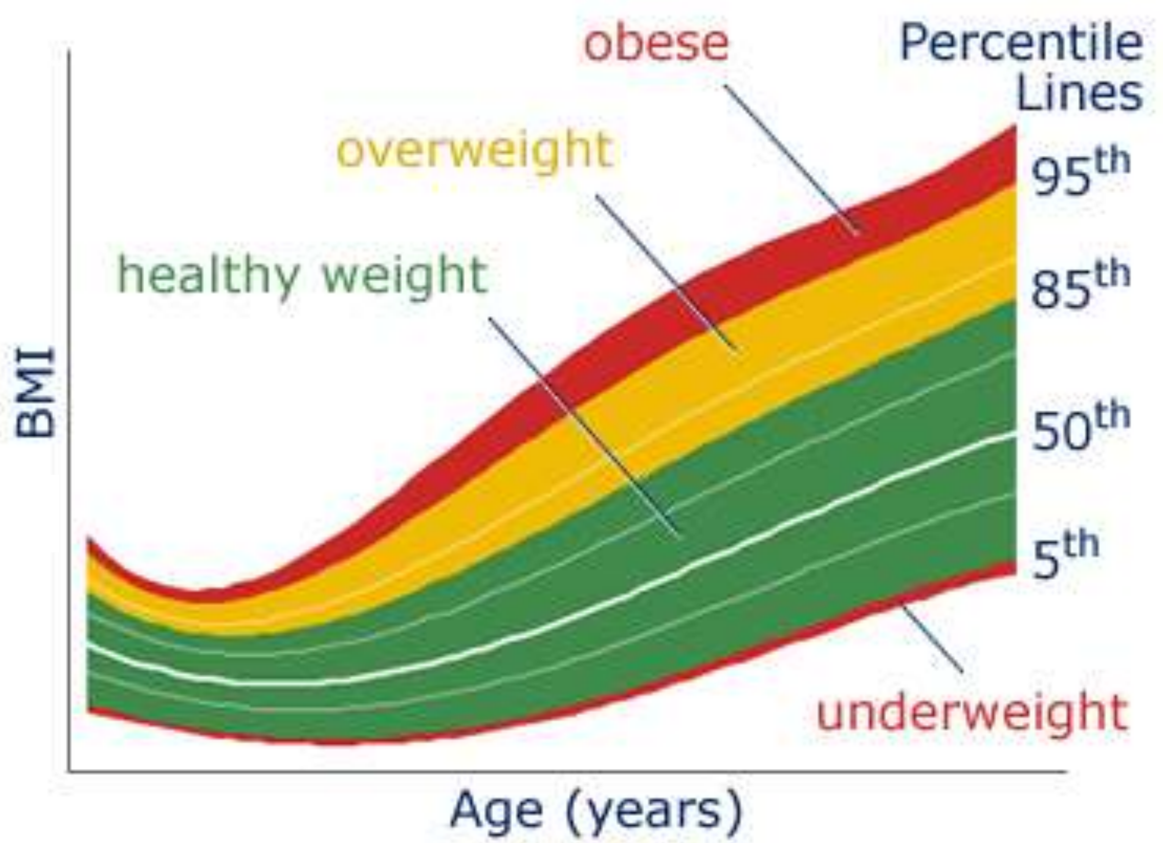

Pediatric Obesity

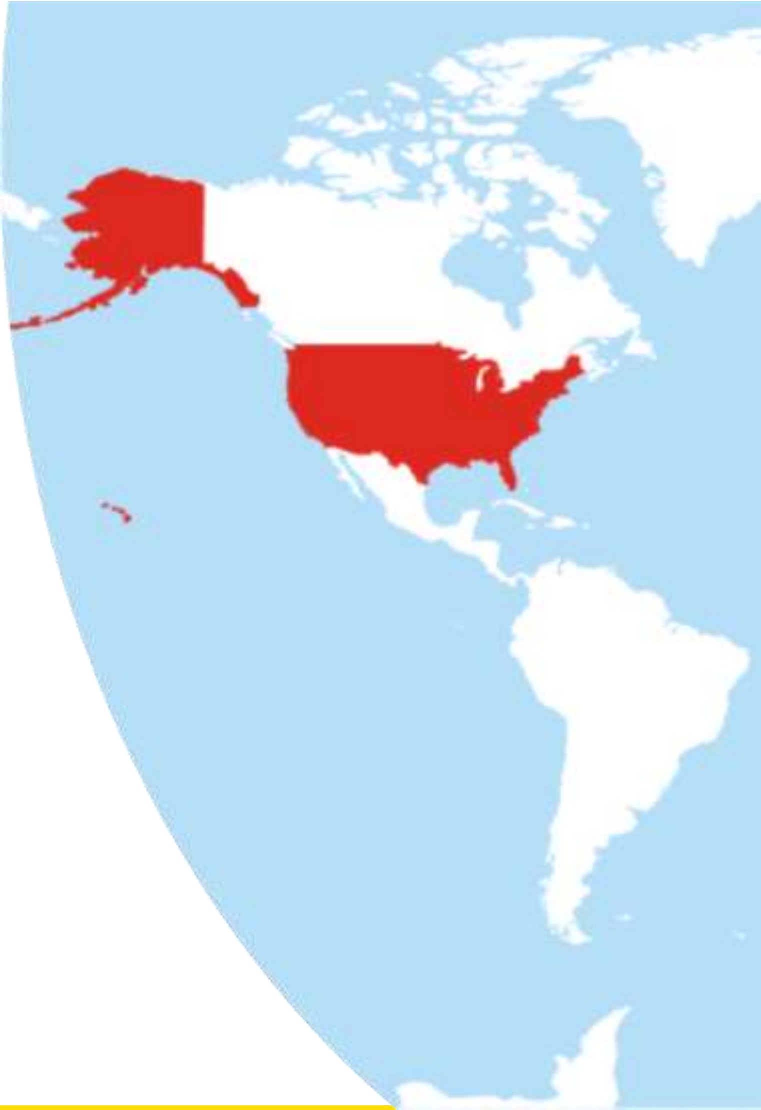

Akron Children's Hospital

Pediatric Obesity

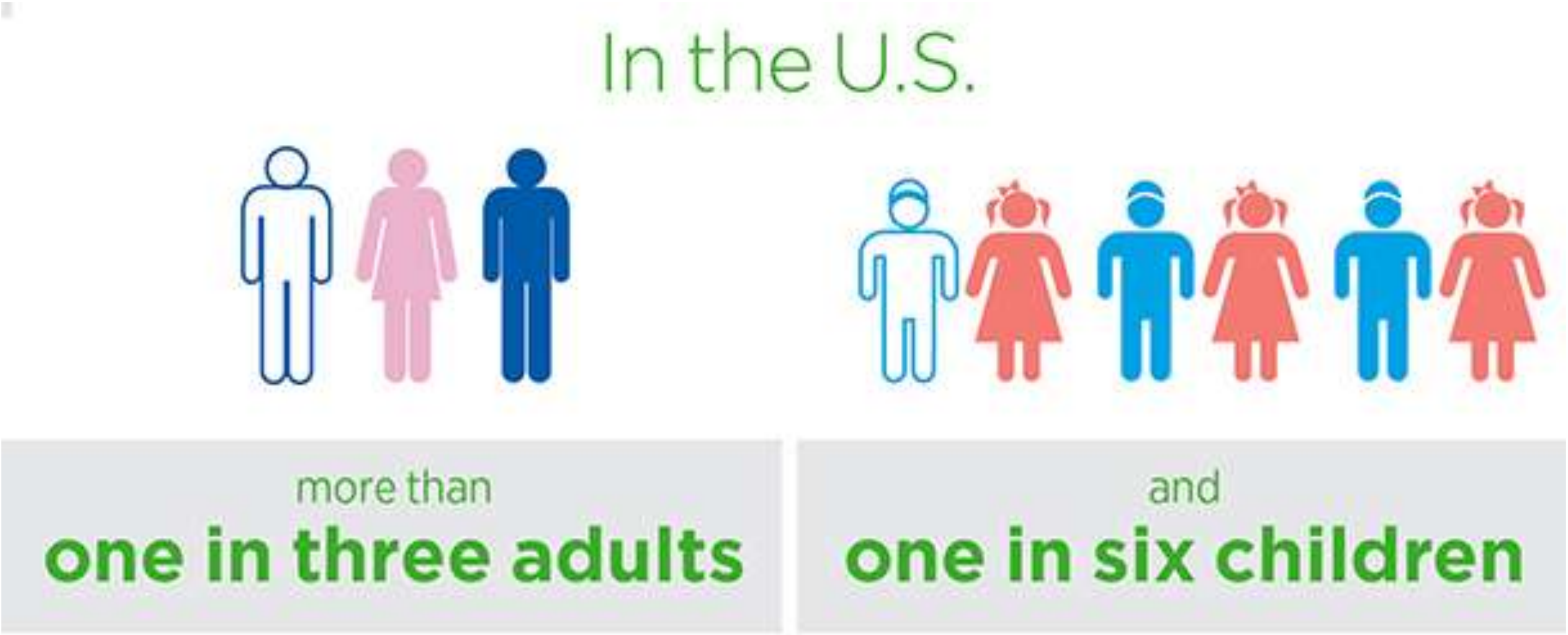

Akron Children's Hosp[illegible]al

Ohio Pediatric Obesity

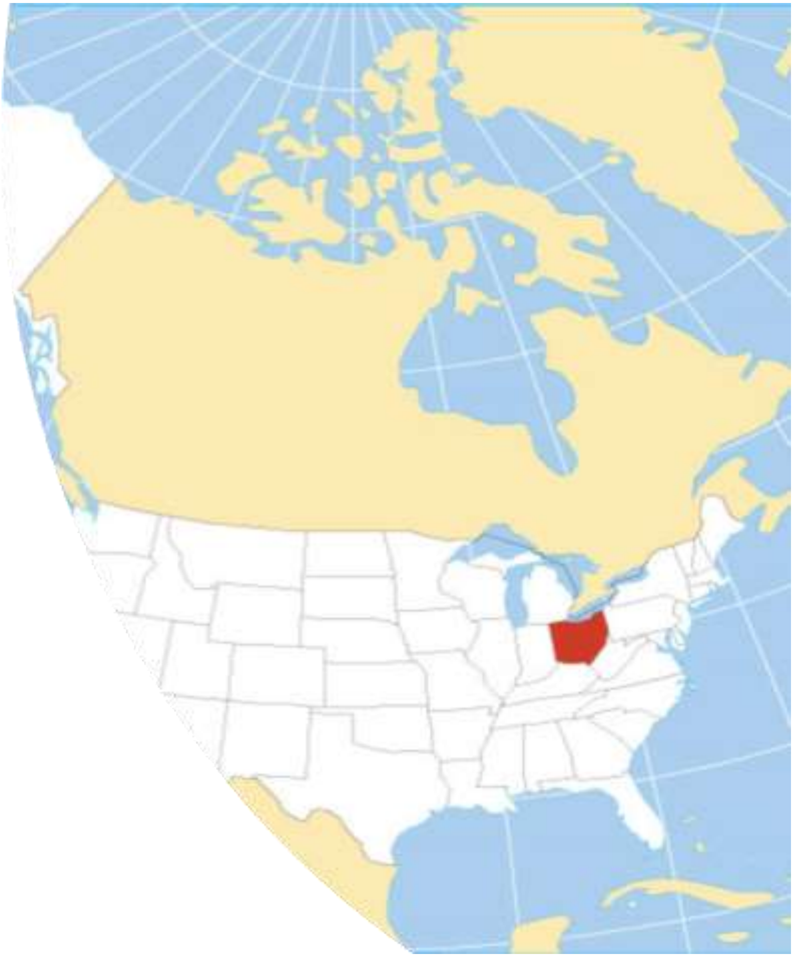

Problem Description

Pediatric Obesity

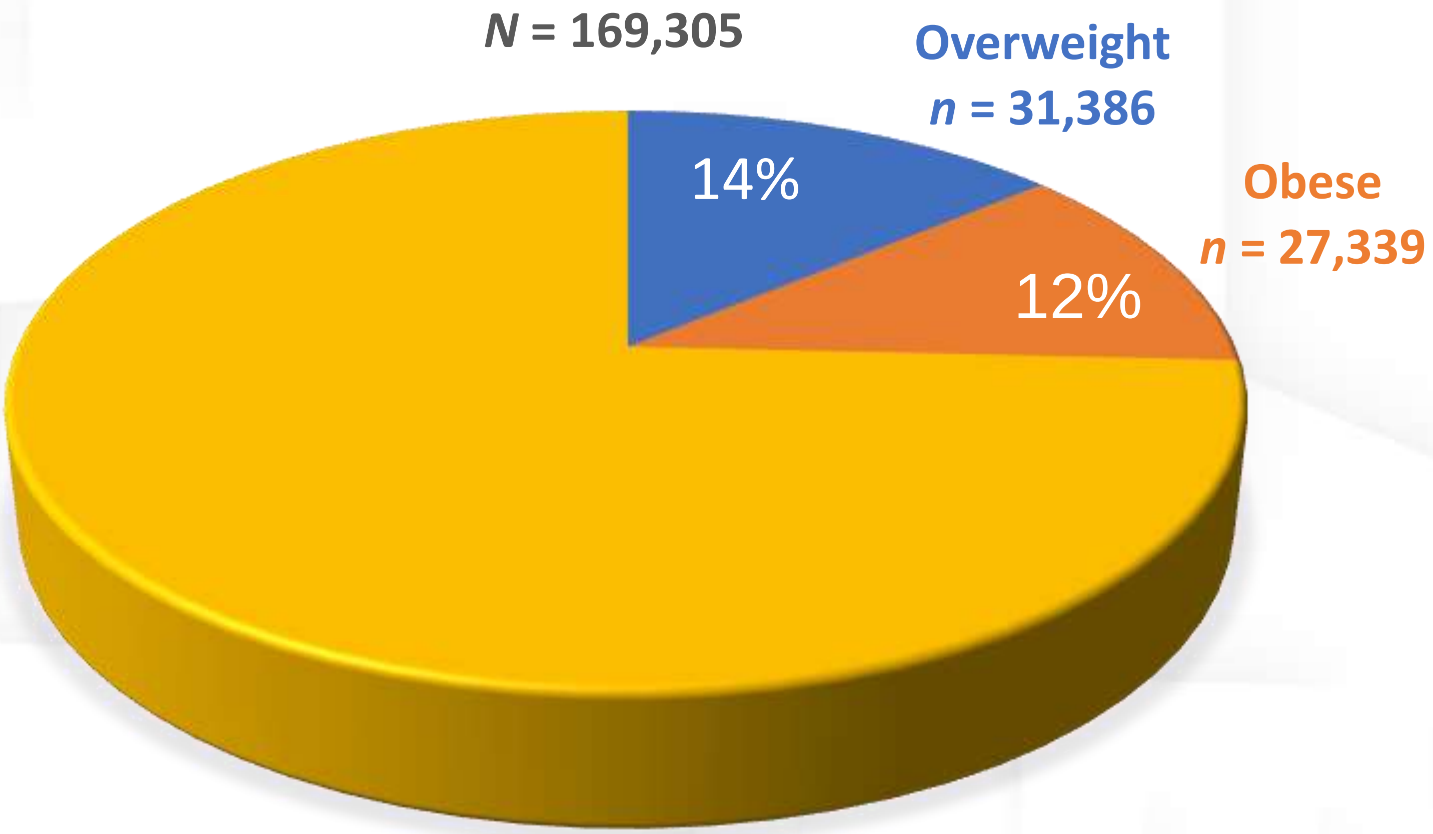

Figure: Primary Care Population Akron Children's Hospital: 2020 (Juszli, 2021; Akron Children's Hospital, 2020).

University of CINCINNATI | COLLEGE OF NURSING

Diseases Associated with Pediatric Obesity

- High Blood Pressure
- High Cholesterol
- Insulin Resistance
- Type 2 Diabetes
- Asthma
- Sleep apnea
- Joint problems
- Fatty liver disease
- Gallstones
- Gastroesophageal Reflux
- Anxiety
- Depression
- Low self esteem
- Bullying
- Social problems

Diseases Associated with Pediatric Obesity

- High Cholesterol
- Type 2 Diabetes
- Fatty liver disease

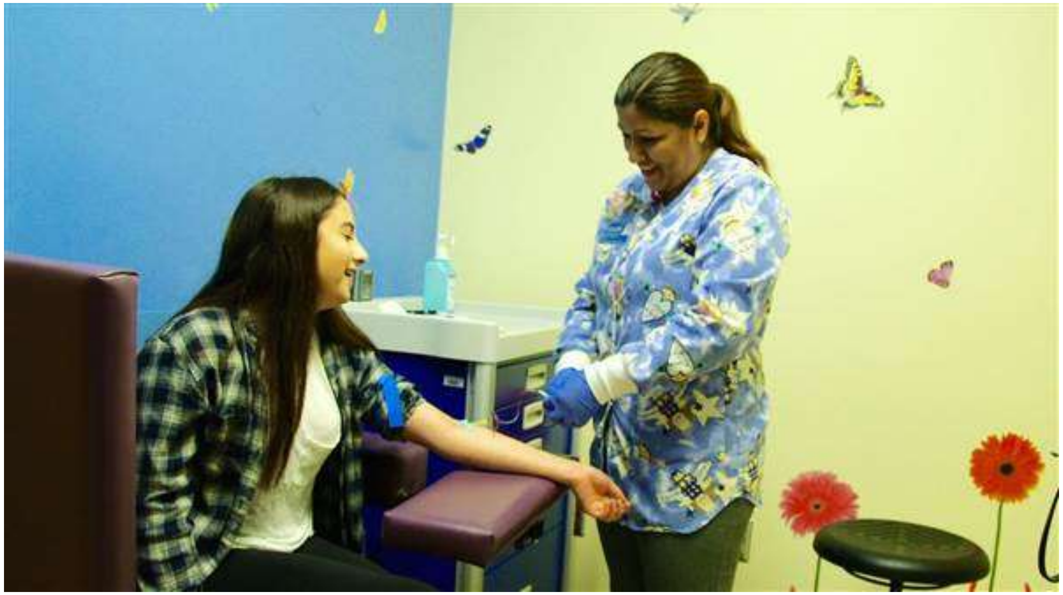

Akron Children's Hospital

✓ **Every 2 years**

- 10 and older with a BMI between 85th-<95th

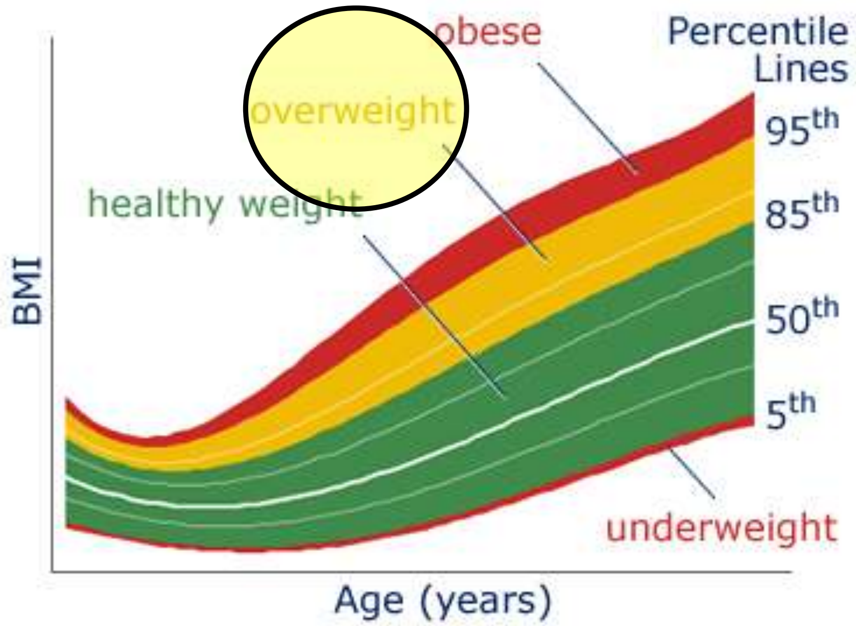

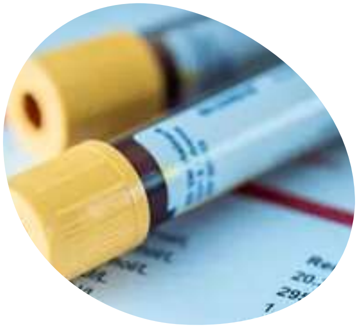

Lab screening

- Lipid panel

✓ Every 2 years

- 10 and older with a BMI between 85th-<95th

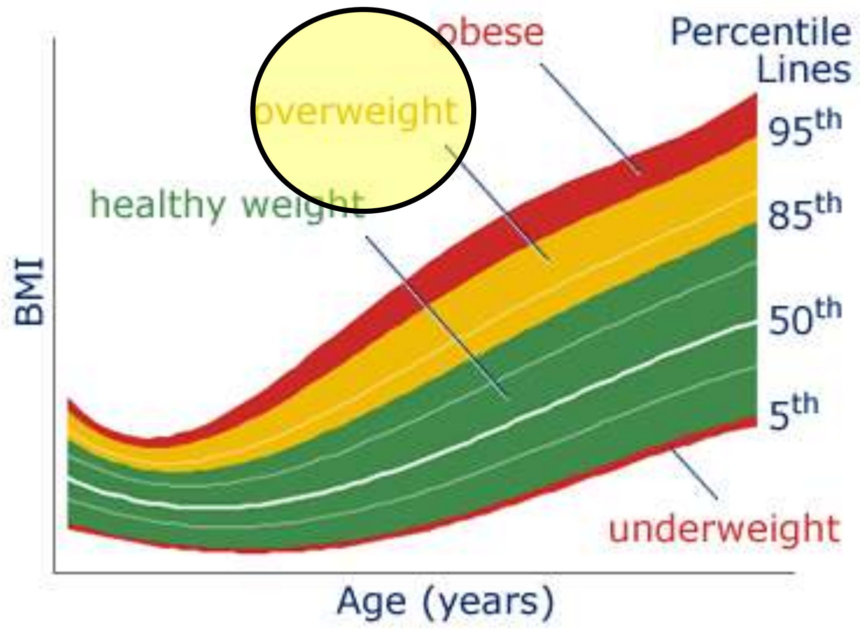

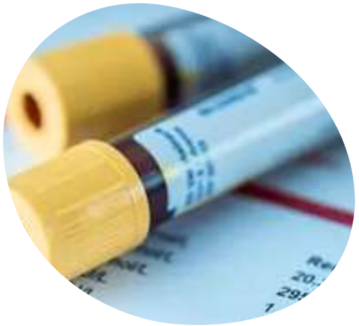

Lab Screening

- Lipid panel
  Risk factors

1st or 2nd degree relative with

- Obesity
- Type 2 diabetes
- Hypertension
- Abnormal lipids
- Heart disease

- Fasting glucose
- ALT

✓ **Every 2 years**

- 10 years and older with a BMI higher ≥95th

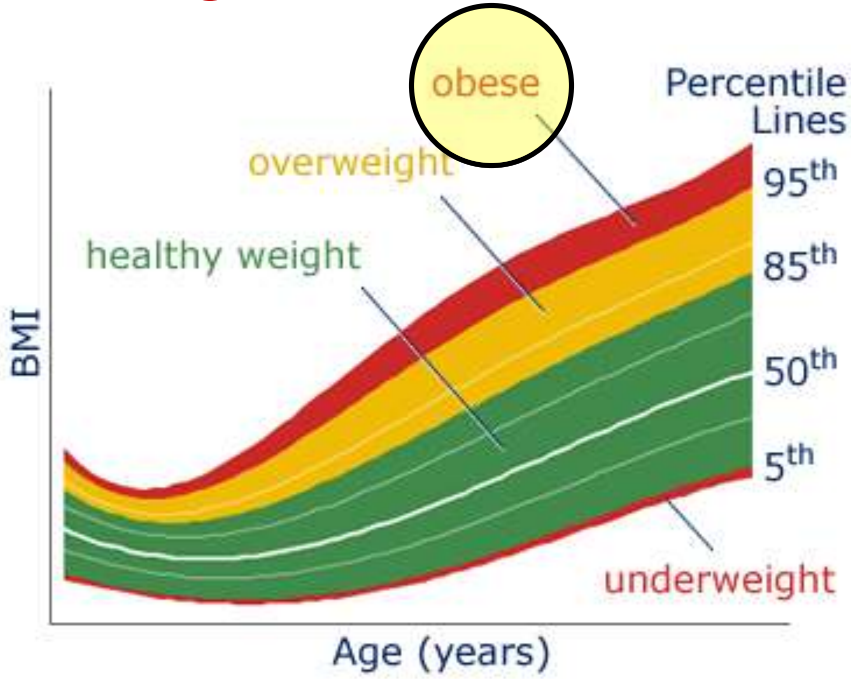

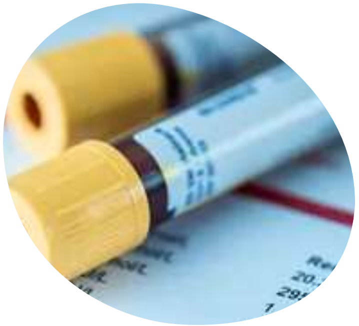

Lab Screening

- Lipid panel
- Fasting glucose
- ALT

Akron Children's Hospital

Low Rates of Recommended Screening Labs

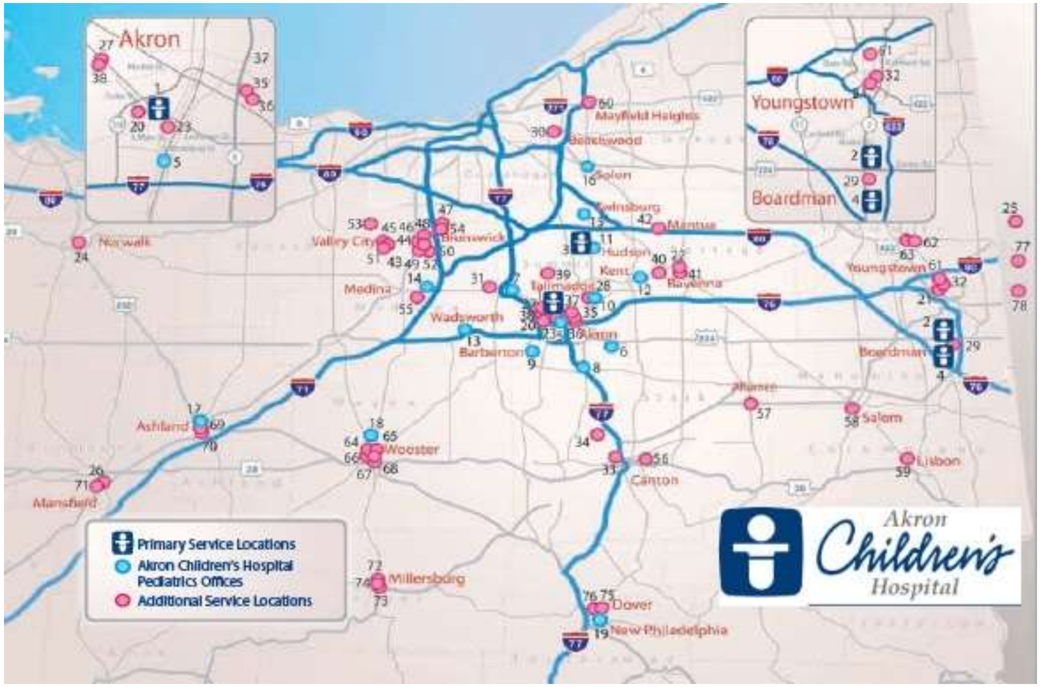

In the United States Screening labs are performed less than 1/3 of the time

Problem Description

Table: Lab Screening in All Primary Care Offices: 2020

Body Mass Index (%)	Labs Completed (%)
85-95	15
95-100	19

N = 809 labs completed for 85%-95%
N = 1648 labs completed for 95%-100%

(Juszli, 2021; Akron Children's Hospital, 2020)

University of CINCINNATI | COLLEGE OF NURSING

Pediatric Comorbid Illness Associated with Elevated BMI

Liver Disease

High Cholesterol

Diabetes

For Providers

Smartsets are already in Epic to help guide lab ordering

Quality Improvement Project Goal

- Increase the percentage of patients 10 years and older with BMI $\geq$95% who have HgA1c, ALT, and lipid panel ordered at well visit if not done in the previous 2 years from 20% to 50% by 12/31/22

For Providers

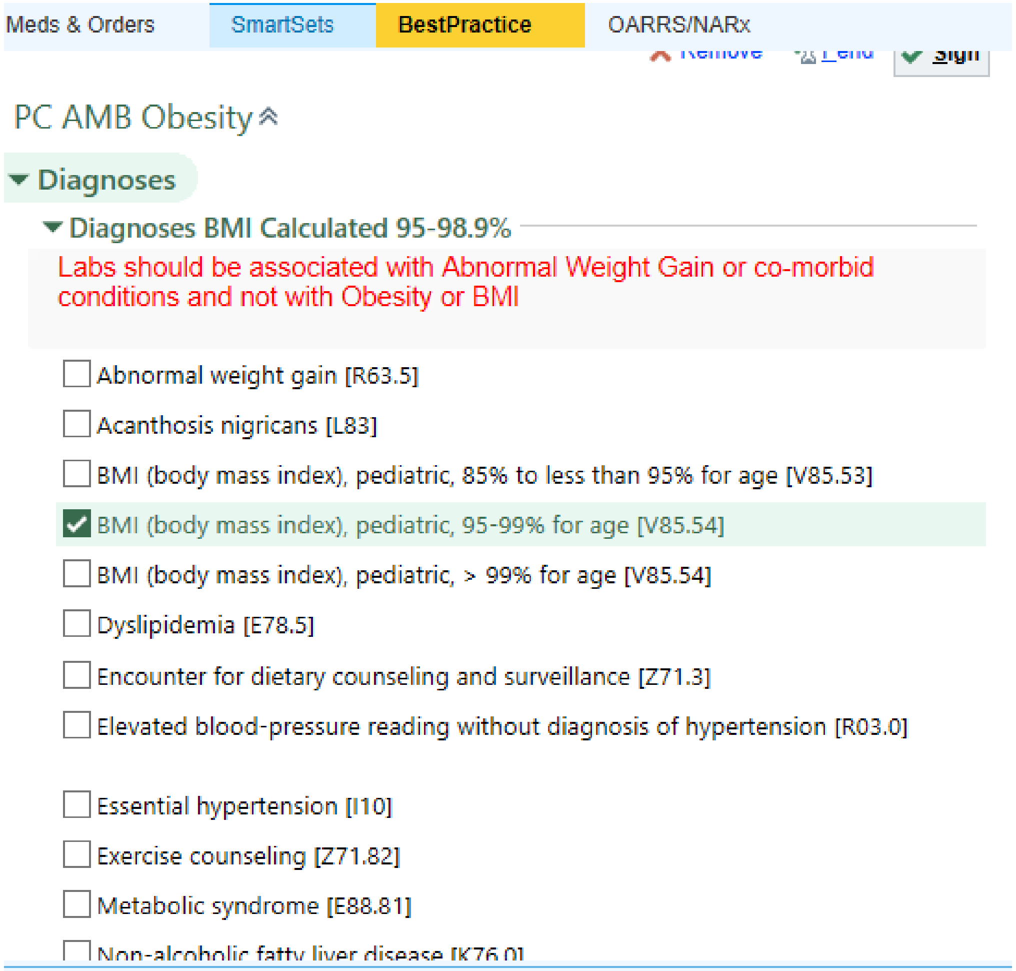

For Providers

Meds & Orders | SmartSets | BestPractice | OARRS/NARx

Clinic Collect Labs - Recommended Labs for Patients with BM...

Labs

ALT/AST and HgbA1c recommended every 2 years. Lipid panel (preferably fasting) if not previously done.

- [ ] Finger/Heel Stick
  Routine, Clinic Performed
- [ ] Venipuncture
  Routine, Clinic Performed
- [ ] POCT Glucose
- [ ] Hemoglobin A1c
  Routine, Clinic Collect
- [ ] AST
  Routine, Clinic Collect
- [ ] ALT
  Routine, Clinic Collect
- [ ] Lipid panel
  Routine, Clinic Collect

Lab Collect - Recommended Labs for Patients with BMI Great...

Labs

For the Healthcare Team

- Lab work for elevated BMI may be performed
  - During the well visit
  - On the Nurse schedule
    - Schedule early in day (fasting)
    - Provider can place future orders within the Smartset
  - At ACH Lab or outside lab (provide printed orders)

Identifying Illness in Children

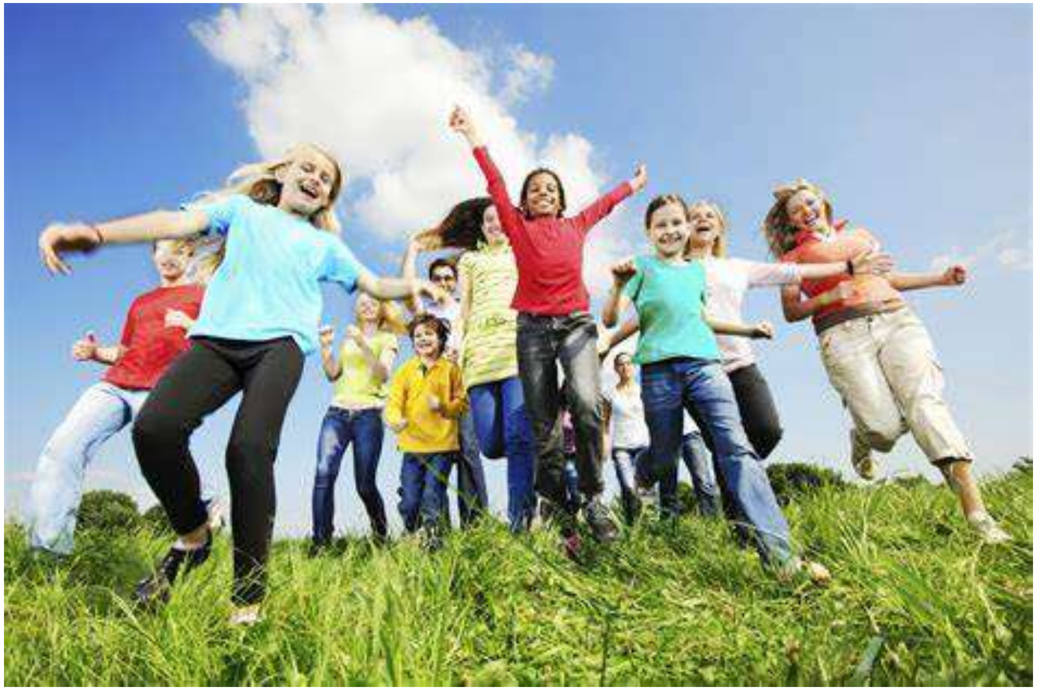

Early treatment of disease

Improved health

Improved well being

Longer life span

Healthier Kids, Healthier Population

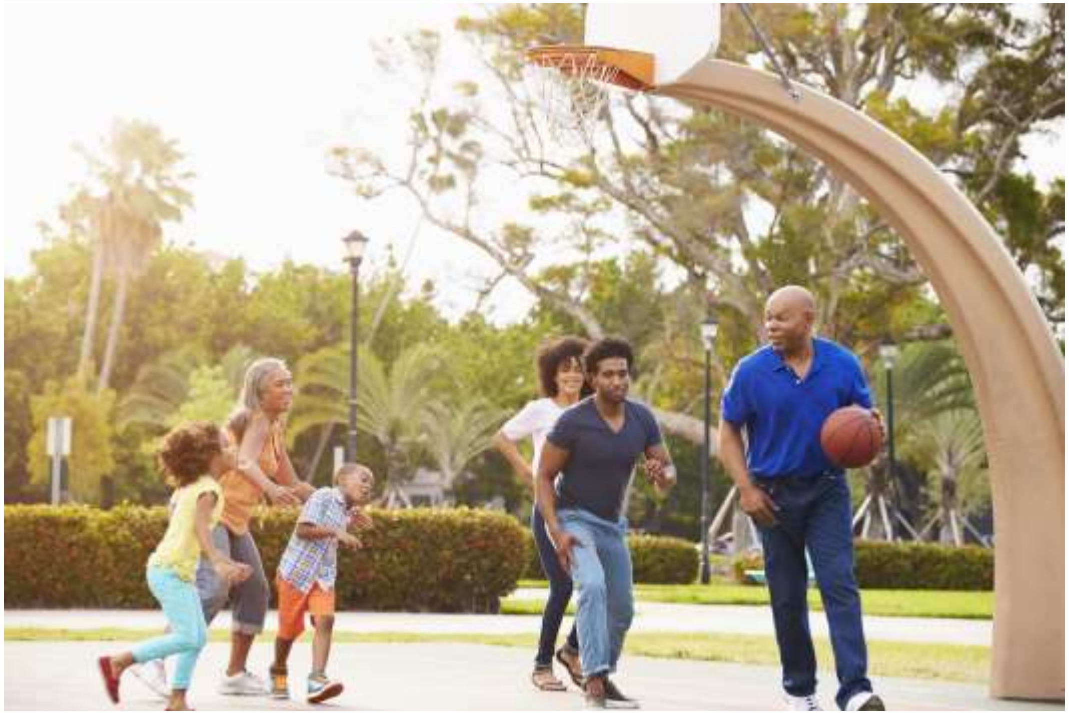

Less adult obesity

Less adult illness

Longer lifespans

Less healthcare costs

Quality Improvement Project

- Providers and operation managers will receive monthly reports of lab orders for children 10 years and older seen for well visit with BMI $\geq$95% or higher

For Providers

- Please print these new and revised documents to help guide the clinical management of children with elevated BMI and abnormal lab results
- Click Here

Thank You for Participating!!!

- Improved Quality
- Improved Care
- ACHP Rocks!!!

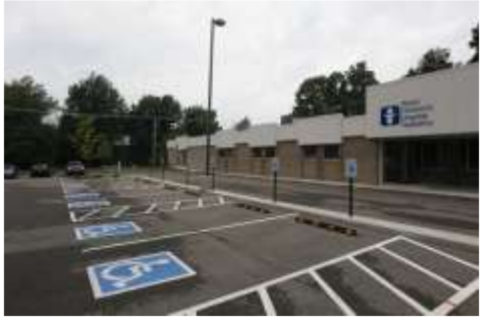

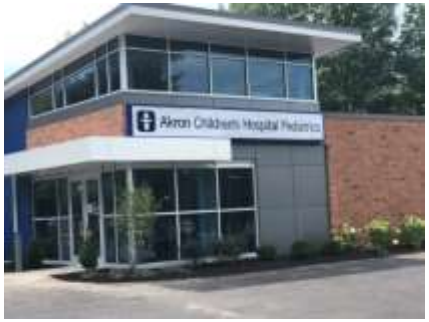

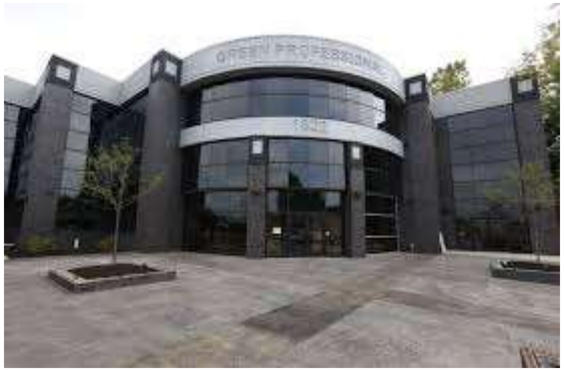

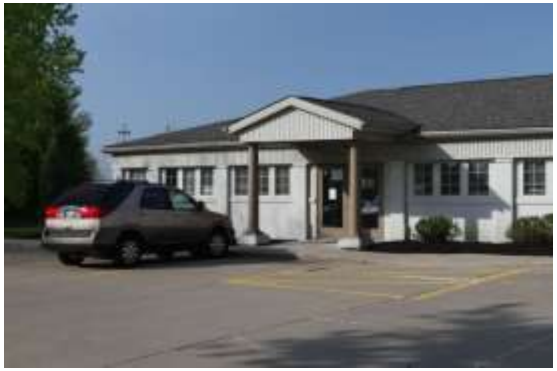

Post Test

- Please click here

Questions?

Sharon Juszli, sjuszli@akronchildrens.org

Dr. Karas, dkaras@akronchildrens.org

Project References
